# Evidence for Follicle-stimulating Hormone Receptor as a Functional Trimer*

**DOI:** 10.1074/jbc.M114.549592

**Published:** 2014-04-01

**Authors:** Xuliang Jiang, David Fischer, Xiaoyan Chen, Sean D. McKenna, Heli Liu, Venkataraman Sriraman, Henry N. Yu, Andreas Goutopoulos, Steve Arkinstall, Xiaolin He

**Affiliations:** From the ‡ EMD Serono Research and Development Institute, Billerica, Massachusetts 01821 and; the § Department of Molecular Pharmacology and Biological Chemistry, Northwestern University Feinberg School of Medicine, Chicago, Illinois 60611

**Keywords:** Allosteric Regulation, Arrestin, G Protein-coupled Receptors (GPCR), Glycoprotein Hormones, Receptor Structure-function, Reproduction, Cysteine-knot Growth Factor, Cysteine-knot Growth Factor

## Abstract

Follicle-stimulating hormone receptor (FSHR), a G-protein coupled receptor, is an important drug target in the development of novel therapeutics for reproductive indications. The FSHR extracellular domains were observed in the crystal structure as a trimer, which enabled us to propose a novel model for the receptor activation mechanism. The model predicts that FSHR binds Asnα^52^-deglycosylated FSH at a 3-fold higher capacity than fully glycosylated FSH. It also predicts that, upon dissociation of the FSHR trimer into monomers, the binding of glycosylated FSH, but not deglycosylated FSH, would increase 3-fold, and that the dissociated monomers would in turn enhance FSHR binding and signaling activities by 3-fold. This study presents evidence confirming these predictions and provides crystallographic and mutagenesis data supporting the proposed model. The model also provides a mechanistic explanation to the agonist and antagonist activities of thyroid-stimulating hormone receptor autoantibodies. We conclude that FSHR exists as a functional trimer.

## Teaser

**Background:** A carbohydrate of follicle-stimulating hormone (FSH) has been proposed to sterically block other FSH molecules from binding to the putative receptor (FSHR) trimer.

**Results:** FSH increases its receptor binding by 3-fold when the steric hindrance is removed.

**Conclusion:** FSHR forms a functional trimer.

**Significance:** This knowledge may improve designs of therapeutic drugs targeting FSHR.

## Introduction

G-protein coupled receptors (GPCR)^2^ are expressed by all types of cells and play a critical role in cellular function and survival. This class of receptors makes up one of the largest families of human proteins and includes the targets of ∼40% of marketed drugs (1). Given the importance of GPCRs in both biology and drug discovery, vast efforts have been made to gain an insight into the underlying mechanisms, leading to many landmark findings. Among the more prominent findings are the cloning of the β-adrenergic receptor (2), the discovery of arrestin regulation (3), and the determination of the crystal structure of the β2 adrenergic receptor-G_s_ protein complex (4). Yet, whereas tremendous progress has been made, there remain many unanswered fundamental questions. In particular, despite the fact that GPCR oligomerization is a well documented phenomenon, it is unclear why GPCRs are fully capable of functioning properly as monomers (5, 6). For some GPCRs, an oligomer is functionally equivalent to a monomer in ligand binding and G-protein activation, as demonstrated for rhodopsin (7) and metabotropic glutamate receptor (8).

The glycoprotein hormone receptors represent a subgroup of GPCRs, including receptors for three gonadotropins, follicle-stimulating hormone (FSH), luteinizing hormone, and chorionic gonadotropin, along with thyroid-stimulating hormone (TSH). These receptors, together with their hormone ligands, play pivotal roles in reproduction, sexual development, and thyroid function. The receptors possess a large N-terminal leucine-rich repeat-containing extracellular domain, which interacts with glycoprotein hormones (GPHs). The binding of GPHs to their respective receptors on target cells activates the G_s_-cAMP-protein kinase A signaling pathway (9, 10). The atomic details of FSH bound to the entire extracellular domain of its receptor (FSHR_ED_) has been reported (11). Surprisingly, the FSHR_ED_ formed a trimer, an unprecedented oligomer form for GPCRs, in the crystal structure. However, it was not clear from these data whether FSHR functions as a trimer in the native state. Intriguingly, such a trimer would provide a rational explanation for several experimental observations, including the important biological roles of the remote-site residues of the GPH, especially the phenomenon that full glycosylation at Asnα^52^ is indispensable for hormonal bioactivity. Furthermore, published articles have shown that electrophoretic bands (12–14), as well as the binding data of FSH to FSHR, as modulated by low-molecular weight (LMW) allosteric modulators (15–18), are consistent with glycoprotein hormone receptor trimers. These observations, in combination with molecular modeling studies, have led us to propose a trimeric glycoprotein hormone receptor activation mechanism (18). The purpose of this study was to design experiments to test the proposed mechanism.

## EXPERIMENTAL PROCEDURES

### 

#### 

##### Cell Culture, Cloning, and Protein Preparation

The procedures of cloning, cell culture, protein expression, and purification were described previously (11). In brief, the coding sequences of FSH and the full ectodomain of human FSHR_ED_ (Ser^16^–Arg^366^) were subcloned into pVLAD6. Initial virus stock was produced by co-infecting Sf9 cells with the constructs and baculovirus DNA. The two viruses were further amplified to co-infect GnTI-HEK293 cells. The recombinantly expressed proteins were captured from the conditioned media and purified with affinity and size exclusion columns. The mutant proteins (αN52D and βT60E) were prepared using the same protocols as the fully glycosylated FSH.

##### X-ray Crystallography

The FSH-FSHR_ED_ complex was concentrated to 10 mg/ml using a disposable ultrafiltration device for crystallization at 20 °C. The P3_1_ crystals were grown from hanging drops mixed 1:1 with a reservoir solution of 0.1 m imidazole, pH 8.0, and 20% Jeffamine M-600. Crystals were cryo-protected with 15% (v/v) ethylene glycol. Diffraction data were collected at the 21-ID-D beam line of the Advanced Photon Source at wavelength of 0.979 Å and processed using the HKL3000 suite (19). The structure was determined by molecular replacement using the FSH-FSHR_ED_ complex (PDB code 4AY9) as the search model (20). Reiterated cycles of model building and refinement were carried out using REFMAC and BUSTER with TLS parameterization (21, 22). The data collection and refinement statistics are shown in Table 1. Structure figures were made using PyMOL.

**TABLE 1:** **Data collection and refinement statistics for the P3_1_ FSH-FSH_ED_ crystal structure** This crystal was grown from a Jeffamine M-600 crystallization solution, different from the PEG4000 buffer used to grow the P1 crystal (11). The Ramachandran allowed region was analyzed by the Molprobilty software (47). Values in parentheses are those for the highest resolution shell.

**Data collection**	
Space group	P3_1_
Wavelength (Å)	0.979
Unit cell (Å, ^o^)	*a* + 95.90, *c* + 204.28
	α + 90, γ + 120
Resolution (Å)	50–2.9 (2.95–2.90)
Completeness (%)	95.5 (70.0)
Mosaicity (^o^)	0.3
Redundancy	8.2 (4.1)
*R*_merge_ (%)	7.9 (25.0)
*I*/σ(*I*)	19.9 (2.5)

**Refinement statistics**	
Resolution range	25–2.5 (2.97–2.90)
No. of unique reflections	42207
*R*-factor (%)	17.4 (25.7)
*R*-free (%)	23.7 (29.1)
Free *R* test set size	5%
No. of non-water/water atoms	11,643/155
Mean *B* value (Å^2^)	86.6
Root mean square deviation bonds (Å)	0.009
Root mean square deviation angles (^o^)	1.21
Ramachandran allowed region	99.3%

##### CHO-hFSHR Membrane Preparation

CHO-DUKX cells expressing the human FSH receptor were disrupted by nitrogen cavitation in a buffer containing 250 mm sucrose, 25 mm Tris, pH 7.4, 10 mm MgCl_2_, 1 mm EDTA, and protease inhibitors (Sigma). The cells were pressurized with 900 p.s.i. of N_2_ gas for 20 min, after which the lysate was centrifuged at 1,000 × *g* for 10 min at 4 °C. The supernatant was then collected and centrifuged at 100,000 × *g* for 1 h at 4 °C. The resulting pellet was re-suspended in binding buffer (10 mm Tris, pH 7.4, 5 mm MgCl_2_) with a Dounce homogenizer. The protein concentration of the samples was determined using the Bio-Rad protein assay reagent.

##### FSH Binding to CHO-hFSHR Membranes

**FIGURE 1.**
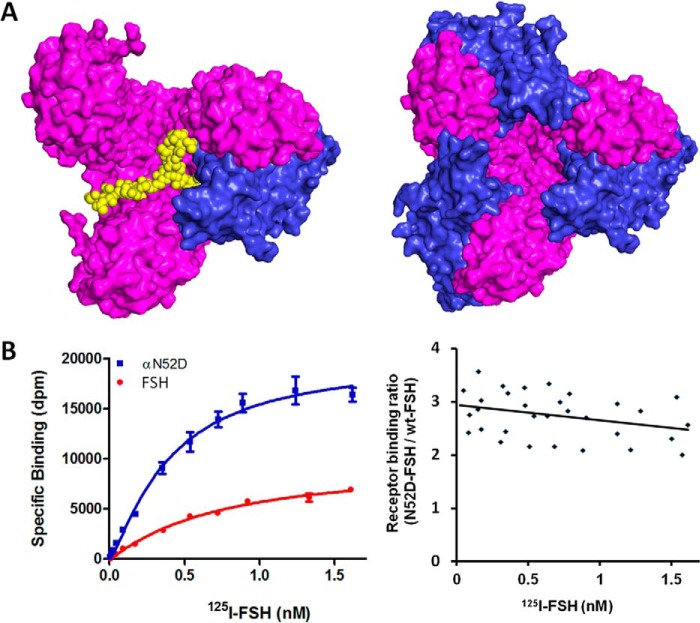
**Effect of FSH glycosylation at Asnα^52^ to its receptor binding.** *A,* spatial consideration of Asnα^52^ glycosylation on FSH binding to its receptor. *Left panel*, a molecular model of a single fully glycosylated FSH molecule binding to an FSHR trimer, viewing from top. For clarity, glycosylations except at the Asnα^52^ site have been omitted. The receptor trimer is shown as a *magenta* surface, FSH amino acids as a *blue* surface, and carbohydrates as *yellow balls. Right panel*, crystal structure of deglycosylated FSH bound to FSHR_ED_ trimer. *B,* experimental validation of the trimeric model prediction. *Left panel*, saturation curves of FSH binding to FSHR. The *curve* represents experiments performed in duplicate samples. *Right panel*, receptor binding ratio of αN52D-FSH mutant *versus* fully glycosylated FSH. An equal amount (5 μg) of cell membrane from the same preparation was used for each derived binding ratio to minimize FSHR count difference. The data reflect the ^125^I-FSH receptor binding assays in four independent assays, each with a different membrane preparation.

**FIGURE 2.**
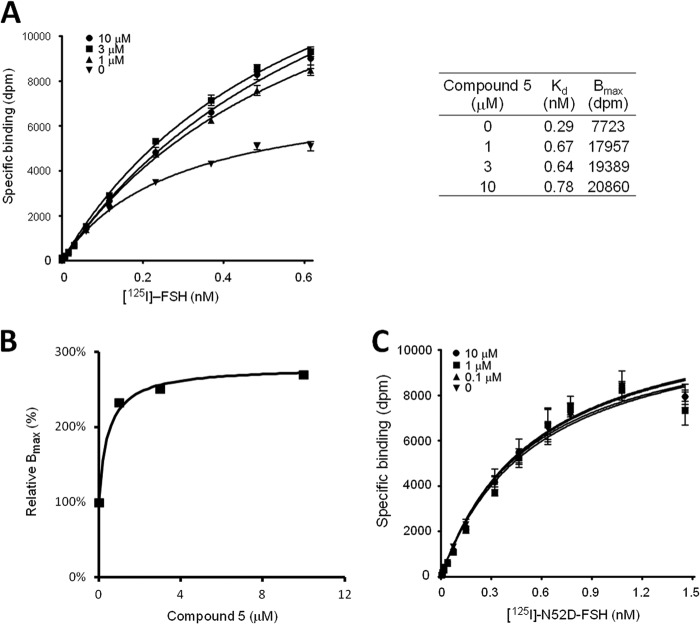
**Effect of LMW allosteric modulators on the FSH/FSHR binding stoichiometry.** *A,* saturation curves of FSH binding to FSHR in the absence or presence of Compound 5 (at indicated concentrations). The *curve* represents experiments performed in duplicate samples. *Right panel*, the FSH *K_d_* and *B*_max_ values at the specified Compound 5 concentration calculated from the saturation curves. *B,* relative FSH binding sites of FSHR at different concentrations of Compound 5 where the *B*_max_ value in the absence of the modulator is normalized to 100%. *C*, effect of Compound 5 on ^125^I-FSH αN52D mutant binding to FSHR. The curve represents experiments performed in duplicate samples.

Radioligand binding assays were performed in 100 μl of 10 mm Tris, pH 7.4, 5 mm MgCl_2_, 0.2% BSA (assay buffer) in 96-well plates (Costar 3365). For the experiments shown in Fig. 1, a fixed amount of 5 μg of CHO-FSHR membrane was mixed with increasing concentrations of glycosylated ^125^I-FSH or ^125^I-N52D-FSH (PerkinElmer Life Sciences). For the experiments shown in Fig. 2, Compound 5 was also added to the membrane at the indicated concentrations. Nonspecific binding was determined in the presence of a 500-fold excess of FSH at each concentration of ^125^I-FSH. The reactions were incubated for 90 min at 37 °C, with shaking, and terminated by filtering through a low protein binding Durapore filter (Millipore Multiscreen), which had been preincubated in assay buffer. The filters were washed 4 times with ice-cold binding buffer (10 mm Tris, pH 7.4, 5 mm MgCl_2_) and counted on a γ counter. Data were analyzed using the GraphPad Prism software.

##### FSHR β-Arrestin Recruitment Assay

**FIGURE 3.**
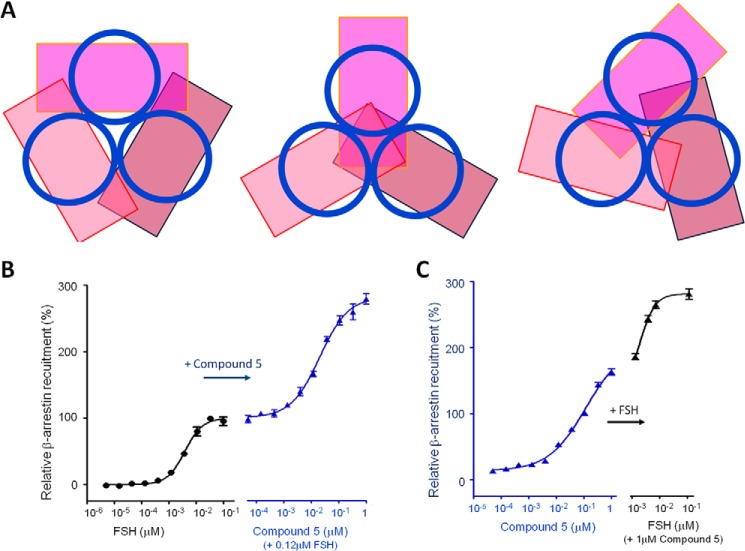
**Effects of Compound 5 on receptor activation.** *A,* consideration of spatial compatibility between a 7-TM domain and β-arrestin. Each 7-TM domain is represented as a *blue circle* and each arrestin as a *magenta-like rectangle*. The *three panels* represent three representative orientations of β-arrestins in relative to the 7-TM domains, assuming a 3-fold rotational symmetry in the 7-TM trimer. It can be concluded that only one β-arrestin can bind to the FSHR trimer due to the steric hindrance along the elongated dimension. *B,* the relative amount of β-arrestin recruited to the activated FSHR inside the CHO cell upon stimulation of FSH alone (*left panel*) or Compound 5 plus FSH at the EC_100_ concentration (*right panel*). The amount of recruited β-arrestin is normalized to 100% for the maximum response of FSH. Data represent experiments performed in duplicate samples. *C,* the relative amount of recruited β-arrestin upon stimulation of Compound 5 alone (*left panel*) or FSH at the EC_20_, EC_50_, EC_70_, and EC_100_ concentrations mixed with 1 μm Compound 5 (*right panel*). Data represent experiments performed in duplicate samples.

**FIGURE 4.**
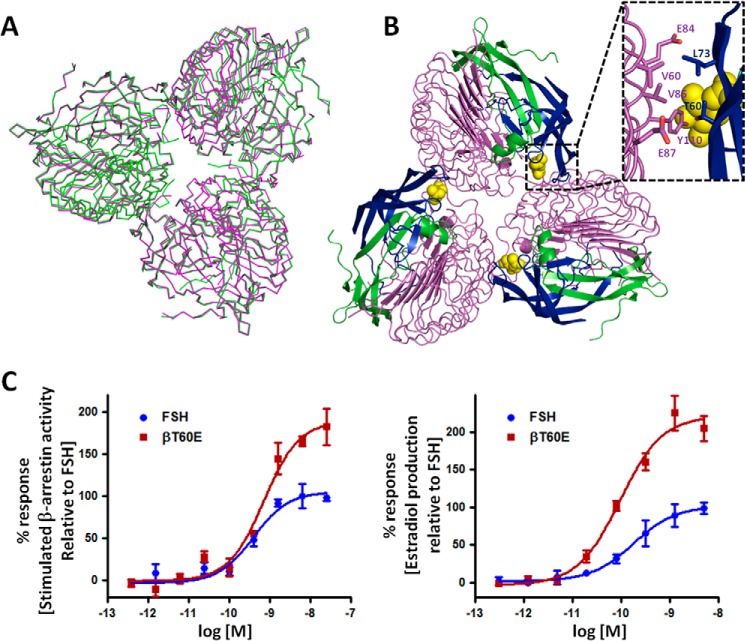
**Crystallographic and mutagenesis studies of the FSH-FSHR complex.** *A,* superimposition of the P1 and P3_1_ trimer structures. P1, *green*; P3_1_, *magenta*. Of 1449 common Cα atom pairs, 1378 pairs were superimposed, resulting in an root mean square deviation of 0.57 Å between the trimers of two space groups. *B,* top view of the trimer observed in the crystal structures. The *inset* shows a close-up view of the potential exosite originating from the FSH-FSHR_ED_ complex oligomerizations. The *magenta ribbons* are for the receptor trimer; *green* and *blue ribbons* are for the FSH α- and β-chains, respectively. The FSH Asnα^52^ glycan is shown as *yellow balls. C,* validation of the roles of the exosite in FSHR activation by FSH mutagenesis. *Left panel*, relative amount of β-arrestin recruited for binding to the activated FSHR upon stimulation by FSH or its mutant. The amount of recruited β-arrestin is normalized to 100% for the maximum response of FSH. Data represent experiments performed in duplicate samples. *Right panel*, relative amount of estradiol production inside primary granulosa cells from immature rats on stimulation by FSH or its mutant. The amount of estradiol production is normalized to 100% for the maximum response of FSH. Data represent experiments performed in triplicate samples.

Determination of activated FSHR was performed by measuring β-arrestin recruitment according to PathHunter FSHR β-arrestin assay protocol (DiscoveRx, product code 93-0517C2) as described previously (11). For the assay shown in Fig. 3, cells were incubated in the presence or absence of FSH and Compound 5 at various concentrations mixed with FSH at an EC_100_ concentration of 120 pm, or FSH at EC_20_, EC_50_, EC_70_, and EC_100_ concentrations mixed with 1 mm Compound 5, respectively. For the assay shown in Fig. 4, cells were incubated with FSH or βT60E-mutant at various concentrations.

##### Primary Granulosa Isolation and Determination of Estradiol Production

Primary granulosa cells from immature rats were used to determine the ability of FSH to induce estradiol secretion, as described previously (11). Briefly, 21-day-old female CD rats were implanted with diethylstilbestrol pellets and euthanized after 72 h for isolation of granulosa cells from the ovaries. The isolated granulosa cells were cultured overnight at 37 °C and subsequently treated with serially diluted FSH for 24 h to determine estradiol production.

##### Molecular Modeling

Homology modeling was performed using the software MOE from the Chemical Computing Group. Structures were analyzed using CCP4 suite software (20), and the conformations were further analyzed against known amino acid conformational tendencies (23). The glycan model in Fig. 1*A* (*left panel*) was constructed by directly linking the bi-antennary glycan in human fibrinogen (PDB entry 3GHG) to an FSH Asnα^52^ residue. The glycan branches were extended to the central cavity of the FSH-FSHR_ED_ complex (PDB entry 4AY9). The other two FSH molecules in the complex were removed due to atomic conflicts. The FSHR 7-TM models were described previously (18). The TSHR trimer model in Fig. 6 was constructed by replacing the FSHR N-terminal residues of PDB entry 4AY9 with the N-terminal structure of TSHR (PDB entry 2XWT) and homology modeling for the TSHR “hinge domain” based on the FSHR crystal structure. The positions of the M22 agonist antibody and K1–70 antagonist antibody were located by superimposing the truncated TSHR in their respective receptor complex structure (PDB entry 3G04 and 2XWT, respectively) onto the TSHR molecule of the TSHR trimer model.

## RESULTS AND DISCUSSION

### 

#### 

##### FSHR Binds Three Times the Amount of Asnα^52^-Mutant-FSH Than Fully Glycosylated FSH

FSH is a glycosylated heterodimer, with four *N*-linked glycosylation sites, two at Asn^52^ and Asn^78^ of the α-subunit and the other two at Asn^7^ and Asn^24^ of the β-subunit. In our trimeric activation model, a constitutive FSHR trimer is only able to bind one fully glycosylated FSH molecule, in contrast to three deglycosylated FSH molecules binding to one FSHR trimer (Fig. 1*A*) (18). This is because the occupied full-length glycan at Asnα^52^-FSH at the central cavity of trimeric FSHR sterically prevents the binding of additional fully glycosylated FSH molecules. Accordingly, our model predicts that the mutant FSH, lacking the glycan at Asnα^52^, would bind to the cell-surface FSHR by a factor of 3 times that of fully glycosylated FSH (18). To test this hypothesis, we mutated FSH residue Asnα^52^ to aspartate (N52D) so that the site would no longer be glycosylated. We then tested the receptor binding capacity of the mutant and compared it to that of glycosylated FSH. The *left panel* of Fig. 1*B* depicts representative data measuring receptor binding of the mutant-N52D and fully glycosylated FSH. The experiment was repeated three times and the binding ratio of the mutant *versus* the fully glycosylated FSH is shown in *right panel* of Fig. 1*B* for all four experiments. The binding ratio fluctuated around 3:1 across a broad range of FSH concentrations, consistent with the hypothesis.

We have considered the difference of binding affinities as an alternative explanation. The explanation was ruled out for three reasons. First, the crystal structures (11, 24) have shown that the glycosylation sites are not in contact with the receptor-binding surface. Second, FSH binds to FSHR at subnanomole affinity. At this high affinity, the ratio of receptor binding between the two forms of FSH would not be higher than 1.5-fold within the tested concentration range, as shown in the calculations in Table 2; therefore, a 3-fold FSH binding is not possible without an increase of binding sites. Finally, in the case of a higher affinity for N52D FSH, it would be expected that lower concentrations would be required to reach receptor binding saturation. However, at the respective saturating doses, the total number of bound fully glycosylated and N52D FSH molecules should be the same, which is not the case.

**TABLE 2:** **Calculated receptor occupancy ratio between two forms of FSH, assuming FSHR exists only as a monomer on the membrane surface** For a ligand-receptor complex at equilibrium, the formula to calculate the percentage of occupied receptor (*O_R_*%) is, *O_R_* % + [*L*]/([*L*] + *K_d_*), where *K_d_* is the dissociation constant and [*L*] is the ligand concentration (48). The determined *K_d_* from curve fitting in Fig. 1*B* is 0.72 and 0.40 nm for fully glycosylated FSH and N52D-FSH, respectively.

[FSH] *nm*	Fully glycosylated FSH (*O_R_*% at *K_d_* of 0.72 nm)	N52D-FSH (*O_R_*% at *K_d_* of 0.40 nm)	Ratio (N52D/glycosylated)
0.5	41%	56%	1.36
1.0	58%	71%	1.23
1.5	68%	79%	1.17
2.0	74%	83%	1.13

We noticed that the ratio of mutant FSH to fully glycosylated FSH appears to drop slightly as the FSH concentration increases. This drop approached but did not reach statistical significance when the data were tested for a statistical significance of a non-zero slope straight line (slope: −0.29; intercept: 2.9; *p* value: 0.06). However, such a drop could be consistent with the fact that fully glycosylated FSHs are heterogeneous regarding the lengths and conformations of carbohydrates, such that at higher FSH concentrations, two smaller glycans with suboptimal conformations may fit into the central cavity of the FSHR trimer. Such a mechanism could explain the negative cooperativity observed for glycoprotein hormones binding to their receptors (25).

##### An Allosteric Modulator Increases FSH Binding 3-Fold

LMW modulators have been observed to increase FSH binding to cell-surface receptors from their normal level by ∼3-fold (15–18). These observations led us to propose that a LMW modulator binds to the FSHR 7-TM domain and induces a conformational change of the receptor. A dramatic conformational change, such as the 14-Å dislocation for the helix TM6 in β2-adrenergic receptor (β2AR) (4), may disrupt the trimeric configuration, resulting in each of the dissociated monomers to bind one FSH molecule. To test this hypothesis, we utilized the LMW FSHR modulator, designated Compound 5 (3-((2*S*,5*R*)-5-(2-((3-ethoxy-4-methoxyphenethyl)amino)-2-oxoethyl)-4-oxo-2-(4-(phenylethynyl)phenyl)thiazolidin-3-yl)benzamide),which has been demonstrated to bind to an allosteric site in the FSHR transmembrane domain (26). We performed ^125^I-FSH binding assays in the presence and absence of Compound 5, by measuring the specific binding of ^125^I-labeled human FSH to the human FSHR. Fig. 2*A* shows the binding data in both the presence and absence of the LMW compound. The maximal binding (*B*_max_) reached 20,860 disintegrations per minute (dpm) in the presence of Compound 5 (at 10 μm), as compared with 7,723 dpm in the absence of Compound 5 (Fig. 2*A*, *right panel*). To reach the ideal state of total separation of trimer, the ratio has to be extrapolated to a maximum concentration of the LMW modulator. The saturated ratio of 2.8 is consistent with the theoretical limit of 3 when every FSHR trimer is fully separated into three FSHR monomers (Fig. 2*B*). Again, we considered the alternative explanation. The 3-fold increase of FSH binding is not due to an increase of ligand affinity. As the calculations in Table 3 show, FSH binding in the presence of the LMW modulator would not be higher than that in the absence of the modulator without an increase of binding sites. Moreover, the number of FSH molecules bound at approaching saturation concentrations in the absence of the LMW modulator is less than in its presence, consistent with a difference in binding site number rather than in affinity.

**TABLE 3:** **Calculated receptor occupancy ratio of FSH binding to FSHR in the presence and absence of Compound 5 (at each concentration), assuming FSHR exists only as a monomer on membrane surface**

[FSH] *nm*	Compound 5 (μm)						
	0 (*K_d_* + 0.29 nm)	1 (*K_d_* + 0.67 nm)		3 (*K_d_* + 0.64 nm)		10 (*K_d_* + 0.78 nm)	
	*O_R_*%	*O_R_*%	Ratio	*O_R_*%	Ratio	*O_R_*%	Ratio
0.5	63%	43%	0.68	44%	0.69	39%	0.62
1.0	78%	60%	0.77	61%	0.79	56%	0.72
1.5	84%	69%	0.82	70%	0.84	66%	0.79
2.0	87%	75%	0.86	76%	0.87	72%	0.82

*^a^ O_R_*%, percentage of occupied receptor.

Furthermore, our model predicts that the LMW modulator should have little effect on the deglycosylated FSH binding to FSHR (18), because all of the binding sites would already be fully occupied. Fig. 2*C* shows the experimental results that confirm the prediction for Compound 5.

##### An Allosteric Modulator Increases FSHR Intracellular Signaling Levels by 3-Fold

To determine whether the increased FSH binding caused by the modulator can increase the level of the associated activation proteins to the receptor, we assessed the level of intracellular signaling immediately following receptor activation. Although several assays are available to measure GCPR activation, G-protein-mediated assays, including ones for cAMP production and [^35^S]GTPγS binding, can lead to an overestimation of the potency and efficacy of compounds in recombinant, overexpressing systems, where different LMW modulators may produce the same maximal response (27). In contrast, the β-arrestin assay can measure GPCR activity with a linear relationship to β-arrestin occupancy (27). In the present study, a β-arrestin recruitment assay was used to assess FSHR activation following stimulation with FSH alone or in combination with Compound 5.

To facilitate the data interpretation, we assessed the theoretical ratio of the FSHR trimer in complex with β-arrestin. Although the β2AR-G_s_ complex structure is known (4), no crystal structure of any GPCR in complex with β-arrestin is available. Fortunately, the crystal structures of active and inactive β-arrestin are available and the major interaction site of β-arrestin with GPCR 7-TM domains is known (28–30). The ratio of the long dimension of a rectangular prism-like shaped β-arrestin to diameter of a 7-TM is ∼2:1, and the 7-TM binds approximately to the center of β-arrestin along the long dimension. This mode of interaction would prevent β-arrestin from binding more than one molecule to the 7-TM trimer, assuming the FSHR trimer possesses a 3-fold or pseudo-3-fold symmetry (Fig. 3*A*). Therefore, we hypothesized that one intact FSHR trimer can only accommodate one β-arrestin. Once the receptor trimer is dissociated, each activated receptor would then be able to bind one β-arrestin molecule. This prediction was tested using Compound 5 to modulate the receptor. As shown in Fig. 3*B*, addition of Compound 5 to a maximally stimulating concentration of FSH (normalized to 100%) resulted in further activation, approaching a plateau of ∼280% of FSH alone.

Compound 5 alone can activate FSHR and recruit β-arrestin to a greater extent than FSH alone (Fig. 3*B*, *left panel*). Addition of glycosylated FSH to a high concentration of Compound 5 resulted in recruitment of β-arrestin at levels approximately to the same 280% of FSH alone (Fig. 3*C*, *right panel*).

##### Crystallographic and Mutagenesis Studies Are Consistent with the Trimer Model

The trimer structure was crystallized from a PEG solution and determined in the P1 space group (11). To test whether trimer configuration depended on the particular crystallization condition, we crystallized and determined the complex structure in the P3_1_ space group from a Jeffamine M-600 crystallization solution. As shown in Fig. 4*A*, the trimeric arrangements are almost identical in both structures, supporting the proposed FSHR constitutive trimer.

Mutagenesis studies also support the FSHR trimer model. As shown in the *inset* of Fig. 4*B*, the FSH residue Thrβ^60^, at the potential exosite of FSH, does not interact with its primary binding monomer but potentially interact with the neighboring monomer. Thrβ^60^ makes hydrophobic contacts with Val^85^ and Tyr^110^ of the neighboring FSHR. A βT60E mutation would then disrupt the hydrophobic interface and create charge-charge repulsion against the neighboring FSHR residue Glu^87^ (Fig. 4*B*, *inset*). The additional disruption of the trimeric interface in the extracellular domains might create enough room for a second FSH to bind, resulting in an enhancement of FSHR signaling. To test this hypothesis, we made the βT60E mutant. The FSH βT60E mutant indeed enhanced signaling in both the β-arrestin and estradiol production assays, as measured by the maximum percent of receptor response (Fig. 4*C*).

##### Proposed Activation Mechanism of the FSHR Trimer

**FIGURE 5.**
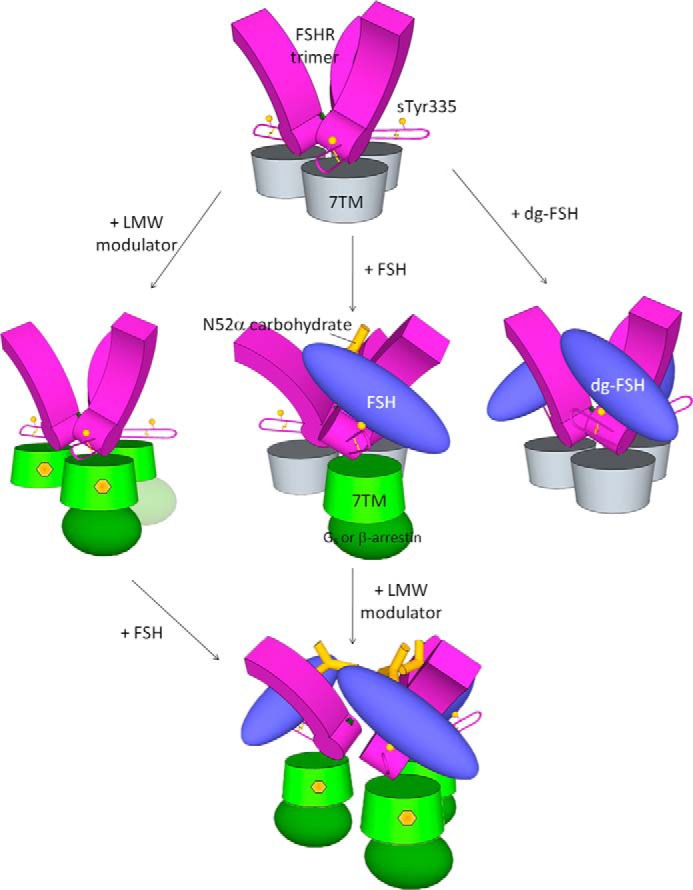
**Proposed mechanism of glycoprotein hormone receptor activation.** The extracellular leucine-rich repeats of the receptor are represented as *purple blocks* with the flexible loops as hairpins and 7-TM domains as *cylinders* (inactivated and activated forms are colored as *gray* and *green*, respectively). The other key receptor elements are also shown, including the sulfate group at Tyr^335^ depicted as *yellow balls*, residues Ser^271^ as *green stars*, and disulfide bonds as *thin yellow lines*. G-protein or β-arrestin are shown as an *ellipsoid*. The GPH heterodimer is shown in *blue*, carbohydrates at Asnα^52^ as *yellow sticks*, and LMW modulators as *yellow hexagons*.

Based on these data, we now further extend our previously proposed activation model for the FSHR trimer (18). For the trimeric receptor to be activated from its extracellular domains, these domains must undergo rearrangement and at least one of the “hinge” hairpin loops has to be shifted. As shown in Fig. 5 (*middle*), FSH normally activates FSHR asymmetrically with the whole trimer acting as a single monomer. Addition of LMW modulators results in the separation of the trimer into monomers. Each separated monomer is then fully functional. Alternatively, FSHR may take a different pathway by activating the 7-TM domains directly with the binding of LMW modulators alone (Fig. 5, *left route*). Depending on the strength of the LMW compound to dissociate the trimer, the number of β-arrestins recruited to one trimeric receptor would vary. As part of an internal screening program targeting FSHR, we have subjected hundreds of LMW hits in β-arrestin recruitment assays, but they alone did not achieve greater than 2-fold of β-arrestin recruitment over the FSH control. This is consistent with the model that insertion of the full-length Asnα^52^ glycan into the central cavity is required for the complete separation of the trimer. Additionally, three deglycosylated FSH molecules can bind to the trimeric receptor (Fig. 5, *right*).

The trimer model may explain the delayed and lower receptor binding of fully glycosylated FSH than the hypoglycosylated FSH, as demonstrated recently (31). In comparison to the fully glycosylated FSH, the hypoglycosylated FSH had a 2-fold increase of receptor binding. The two forms also differ in their receptor binding behavior. The fully glycosylated FSH displayed a sigmoid binding curve, with a slow start in the first 50 min, followed by a rapid period of 2 h before it reached the saturation level. In contrast, hypoglycosylated FSH showed a hyperbolic curve, with almost no signs of delay. Because deglycosylation does not significantly change the amino acid structure of glycoprotein hormones (18, 32–35), the glycans must be attributed to the hampering kinetic effect of receptor binding. It is unclear, however, which of the four glycans plays the most important role, due to the fact that both α and β chains of their hypoglycosylated FSH heterodimer are less glycosylated. The Asnα^52^-glycan of their hypoglycosylated FSH adopts a compact helical shape, due to its higher mannose content than that in the fully glycosylated FSH (31). The smaller and compact glycan at Asnα^52^ in the hypoglycosylated FSH would readily fit into the central cavity of the FSHR trimer, rendering a smooth, hyperbolic binding curve. In contrast, the more bulky and extended glycan of the fully glycosylated FSH would require more time to fit into the central cavity, resulting in a delayed, sigmoid binding curve. Finally, the central cavity can also accommodate more of the compact glycan, allowing more hypoglycosylated FSH binding to the receptor trimer.

The trimer model is also consistent with the observation that ligand binding is increased for TSHR or FSHR with the constitutively active mutation D6.30G (*i.e.* D619G and D567G, respectively). Several constitutively active mutations in the TSHR 7-TM domain cause increases in TSH binding (36). Among these mutants, the D6.30G mutation is most interesting. This negatively charged residue is well conserved in GPCR family members. The equivalent residue (E6.30, *i.e.* Glu^268^) in β2AR plays a central role in receptor activation (37), and moves dramatically during the activation (14 Å outward from the inactive state) without causing significant conformational changes for the residues in the top half (*i.e.* toward the extracellular side) of the 7-TM domain (4, 38). When normalized for receptor number expressed on live cells, a D6.30G mutation of TSHR and FSHR resulted in a 3-fold increase in TSH and FSH binding, respectively (36). Although without further investigation a change in ligand affinity caused by this mutation cannot be formally ruled out, the low likelihood of this mutated residue causing a change in the binding affinity of the anti-TSHR or FSHR antibody used to normalize receptor number, together with the fact that the mutation occurs in a site with the potential to destabilize receptor oligomers, are consistent with our FSH:FSHR binding model.

##### Remaining Open Questions

The proposed mechanism postulates the existence of FSHR as a functional trimer in the native state, which has not yet been demonstrated by direct evidence. Direct evidence might come from the crystal structure of a full-length FSHR in the ground state, an electron or atomic force microscope image of FSHR on a membrane surface, or a super-resolution single-molecule optical image on a live cell. All of these approaches would require specialized capabilities. Nevertheless, the observed electrophoresis band of molecular mass ∼240 kDa in harsh SDS-containing solutions (12) does support the existence of strong FSHR trimers in the ground state. It is not unprecedented for a membrane protein to exist exclusively as a non-covalently linked oligomer in both the native functional form as well as in the presence of SDS, as shown for SKC1 (39, 40).

The proposed model also does not address the mechanism of how the binding of LMW modulators causes the conformational change of the 7-TM domains that leads to subsequent separation to monomers. Although receptor activation is known to change the 7-TM conformation dramatically (4), it is unclear how an FSHR antagonist (ADX68692) also increased FSH binding by 3-fold (41), whereas a partial agonist (Org 42599) was ineffective in the binding increase (17, 42). As LMW modulators can bias FSHR activation (43), the details of the conformational changes upon bindings of the LMW modulators await the crystal structures of such modulators bound to the 7-TM domains.

Earlier reports did not explicitly note a 3-fold increase of FSH binding to FSHR by allosteric modulators (15–17), nor was the mechanism of action consistent with the model proposed herein. Rather, in these studies, the increased binding was attributed to tighter receptor affinity or to enhanced receptor expression. The results in the current report demonstrating a 3-fold increase in binding of FSH to FSHR by Compound 5 cannot be explained by either increased intrinsic affinity or enhanced expression, as (i) no increase in ligand binding affinity was observed, and (ii) current studies were performed on receptor-expressing membranes rather than on viable cells. In the absence of a direct comparison between Compound 5 and other modulators in our model system, it remains speculative as to whether the earlier tested modulators mediate different mechanisms of action. However, it should be noted that the presence of Org 214444 resulted in a 2-fold increase in *B*_max_ at 1 μg but no results were reported at higher concentrations (17). More studies on LMW modulators will be needed to understand the details of the receptor activation mechanism, as called for recently (41).

Until the crystal structures of the full-length FSHR in free form and in complex with LMW modulators are available, it is an open question whether the mechanism of action is truly caused by conformational changes in the 7-TM domains; thus, the proposed model remains a work in progress. Despite these uncertainties, this trimer model may help stimulate new ideas and motivate new research in this field.

##### Implication for the Mechanism of TSHR Autoantibody Agonist and Antagonist Activities

The TSHR is a major autoantigen in autoimmune thyroid disease. Two types of TSHR autoantibodies have been discovered. Antibodies with thyroid-stimulating (agonist) activity are responsible for the hyperthyroidism of Graves disease, whereas antagonist antibodies cause hypothyroidism by preventing the binding of TSH to TSHR. Unlike TSH, which requires sulfation of tyrosine 385 on TSHR for receptor activation, the tyrosine is not required for stimulating autoantibodies to activate the TSHR (44). It was expected that the binding of the stimulating autoantibodies would cause a conformational change in the TSHR. The crystal structures of the hormone-binding portion of TSHR (amino acids 22–260) (TSHR260) in complex with a Fab fragment of thyroid-stimulating autoantibody (M22) (PDB entry 3G04) and a blocking type TSHR antoantibody (K1–70) (PDB entry 2XWT) were determined (45, 46). These crystal structures showed no conformational difference for that portion of TSHR. Thus, how the binding of M22 causes receptor activation has remained poorly understood (46).

**FIGURE 6.**
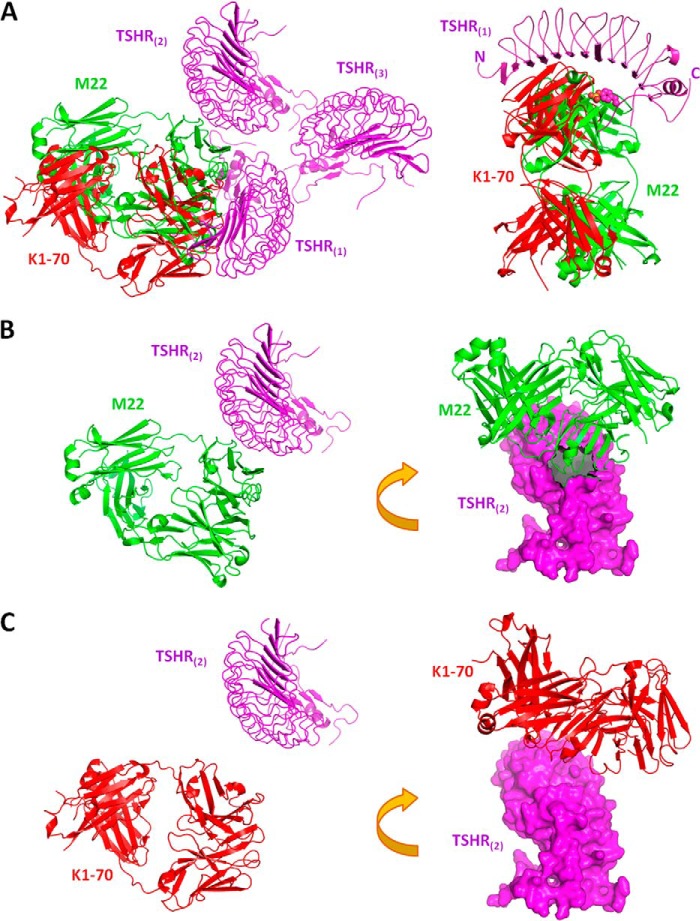
**Explanation of TSHR autoantibody agonist and antagonist activities.** *A*, theoretical model of the TSHR extracellular domain (*TSHR_ED_*) in complex with TSHR autoantibodies, M22 and K1–70. The molecules are shown as color-coded ribbons, marked by their names in the corresponding colors. *Left panel*, the TSHR trimer:autoantibody model. For clarity, only one TSHR protomer of the trimer is shown to bind the antibodies. *Right panel*, the TSHR monomer-autoantibody model. The N- and C-terminals of TSHR ectodomain are marked by their respective *letters*. The hinge sulfated tyrosine side chain is shown as *colored balls. B,* M22 agonist autoantibody clashes with its neighboring TSHR. *Left panel*, same orientation as in the *left panel* of *A. Right panel*, a rotated orientation to show the clashed surface (∼300 Å^2^). TSHR_(2)_ is shown in a *magenta* surface. *C,* same representation as in *B* except the autoantibody is K1–70. Note that there is no clash between the autoantibody and its neighboring TSHR.

Our model may explain the activities of these two different classes of autoantibodies. As both the stimulating antibody M22 and the blocking antibody K1–70 bind to nearly identical epitopes on the concave surface of TSHR, the resulting opposite bioactivities have been difficult to explain. However, as the bound antibodies have differing orientations, corresponding to a rotation of ∼155° along their respective longitudinal axes (46), the M22 (*green* colored), but not K1–70 (*red* colored) clashes with the hinge hairpin loop in the current model (Fig. 6*A*). Therefore, M22 would have shifted the hinge hairpin loop on the 7-TM domain. Consistent with our model, such a shift, caused by an antibody or a ligand, would be critical in the activation of the GPCR.

As our trimer model suggests, and the deglycosylated hormones demonstrate, the ligand-hairpin loop interaction constitutes one of the two requirements in receptor activation via the extracellular domain. The other requirement is the disruption or perturbation of trimeric configuration of the extracellular domains. According to our model, the agonist M22 clashes with its neighboring receptor TSHR_(2)_, whereas the antagonist K1–70 does not (Fig. 6, *B* and *C*). The clashing area of M22 on TSHR_(2)_ is 300 Å^2^ (Fig. 6*B*, *right panel*). If we assume TSHRs adopt the same trimeric configuration as that of the FSH-FSHR_ED_ complex, M22 binding would encounter steric hindrance, pushing the neighboring receptor aside. Indeed, steric hindrance to thyroid-stimulating antibody binding to the TSHR on the cell surface was observed (49). Essentially, the mechanism by which M22 activates the TSHR mimics that of FSH to FSHR, dislocating the hairpin loop and disturbing the trimeric configuration. In contrast, K1–70 does neither of these two actions.

##### Closing Remarks

The central piece of the proposed hypothesis is the existence of FSHR as a constitutive trimer, which is normally capable of binding a single fully glycosylated FSH, leading to the activation of a single G protein and binding of β-arrestin. The results from our designed experiments confirm the predicted 3:1 stoichiometric ratio based on the receptor binding of Asnα^52^-deglycosylated FSH *versus* the fully glycosylated FSH, the binding of FSH and subsequent β-arrestin recruitment following stimulation in the presence and absence of a LMW modulator, and by mutagenesis studies demonstrating that disruption of the hydrophobic interaction at the FSH exosite enhances receptor stimulation efficacy. The model is further supported by our crystallographic studies that reveal that the FSHR trimeric structural configuration is not dependent on the crystallization conditions and space groups (as in the cases of P1 and P3_1_). As GPCR oligomerization may be a general phenomenon, conclusions from our studies may shed light on the activation mechanism of other oligomeric GPCRs. The knowledge of the FSHR activation mechanism may be used in improving therapeutic drugs targeting FSHR and the related receptors.

## Footnotes

2 The abbreviations used are:

GPCR: G-protein coupled receptor
7-TM: seven transmembrane
β2AR: β2-adrenergic receptor
GPH: glycoprotein hormone
LMW: low molecular weight
TSH: thyroid-stimulating hormone
PDB: Protein Data Bank.
